# Alleles of *Chaser*, a dominant modifier of the *Drosophila melanogaster foraging* gene, are consistent with variegating alleles of the heterochromatic gene *spookier*

**DOI:** 10.1093/genetics/iyaf123

**Published:** 2025-06-26

**Authors:** Alistair B Coulthard, Richard Yuditskiy, Alexander R Molnar, Qaim Ali Ramazan, Marla B Sokolowski, Arthur J Hilliker

**Affiliations:** Department of Biology, York University, Toronto, ON M3J 1P3, Canada; Department of Biology, York University, Toronto, ON M3J 1P3, Canada; Department of Biology, York University, Toronto, ON M3J 1P3, Canada; Department of Biology, York University, Toronto, ON M3J 1P3, Canada; Department of Ecology and Evolutionary Biology, University of Toronto, Toronto, ON M5S 3B2, Canada; Child and Brain Development Program, Canadian Institute for Advanced Research (CIFAR), Toronto, ON M5G 1M1, Canada; Department of Biology, York University, Toronto, ON M3J 1P3, Canada

**Keywords:** *Chaser* gene, larval foraging behavior, cGMP-dependent protein kinase, *foraging* gene, ecdysone, heterochromatin

## Abstract

The relationship between genes and quantitative behavioral traits involves complex regulatory networks. Identifying genes that operate in these regulatory pathways can be challenging, especially when dealing with dominant genetic factors. Our work has focused on a naturally occurring behavioral polymorphism in larval foraging behavior in *Drosophila melanogaster*. This polymorphism in larval foraging behavior arises from variation in the *foraging* (*for*) gene with its rover and sitter naturally occurring variants. The dominant rover allele (*for^R^*) results in larvae which move longer distances while foraging compared with larvae with the recessive sitter (*for^s^*) alleles. In this article, we report the successful mapping of the *Chaser* (*Csr*) gene, a dominant modifier of larval foraging behavior which makes sitter larvae behave in a rover-like manner. We localized *Csr* by first mapping recessive phenotype tags closely linked to *Csr*. These phenotype tags mapped to the centromeric heterochromatin on the right arm of chromosome 3. We showed through a combination of deletion mapping, qRT-PCR and feeding of ecdysone hormone to larvae during development that the alleles of *Csr* are consistent with variegating alleles of the gene spookier (*spok*). With *spok* being an essential gene in the synthesis of the molting hormone ecdysone, we have established a link between ecdysone signaling and larval foraging behavior in *D. melanogaster*.

## Introduction

Forty years ago, localizing genes for quantitative behavioral traits by conventional recombination mapping was a daunting task because environmental variation, genetic background, and genetic markers used in recombination mapping modify the expression of wild-type differences in behavior. To address this, de Belle *et al*. (1989) developed “lethal tagging”, a method used in conjunction with deficiency mapping, to localize major genes associated with quantitative traits in *Drosophila melanogaster*. The lethal tagging method was used successfully to show that the *foraging (for)* gene influences wild-type variation in larval foraging path-lengths and that *for* was synonymous with the *dg2* gene, a cGMP-dependent protein kinase (PKG) (de Belle at al. 1989, 1993; Kalderon and Rubin 1989; Osborne *et al*. 1997; Allen *et al*. 2017).

The lethal-tagging approach used mutagenesis to generate mutations in and near the gene responsible for the path-length phenotype. The screen looked for mutations that changed the behavior of the genetically dominant rover larvae, which move longer distances while foraging, into sitter-behaving larvae, which move shorter distances while foraging. The screen also looked for shared recessive pupal lethality (the lethal tag). The shared lethal tag would be closely linked to the gene affecting rover-sitter path lengths, allowing the mapping of the gene responsible for the rover-sitter path-length differences based on lethal complementation analyses of the lethal tag. Subsequent recombination and deletion mapping localized the lethal tag to cytological region 24A3-5 (de Belle *et al*. 1989, 1993). Eventually, *for* was revealed to be synonymous with *dg2* (Osborne *et al*. 1997) where transgenic increase of *dg2* using *dg2*-cDNA in a sitter produced rover larval path-lengths and rover whole larvae had higher *for/dg2* mRNA levels and PKG enzyme activities than sitters (Kaun *et al*. 2007). More recent work showed that *for* has a complex gene structure with 4 promoters (pr1-pr4), 21 transcripts and 8 protein isoforms (Allen *et al*. 2017; Sokolowski *et al* 2023; Vasquez *et al* 2023). Rover and sitter larvae differ in messenger RNA (mRNA) levels from pr1 with rovers having significantly higher mRNA expression in their dissected central nervous systems than sitters (Dason *et al*. 2020). In contrast, rover and sitter larval dissected central nervous systems do not differ in mRNA levels from pr2, 3 or 4. The expression of *for* transcripts in *for* null larvae using a *for*-pr1-GAL4 results in rover-like path-lengths (Allen *et al*. 2018). These data support the notion that rover-sitter differences in larval path-length arises from differences in their gene expression.

The lethal-tagging method was also used in Pereira *et al*. (1995) to identify the *Chaser (Csr)* gene, which has a dominant effect on larval path-length; *Csr* changes sitter-behaving larvae into rover-behaving larvae. The lethal tagging of *Csr* involved mutagenizing sitter flies, crossing them to the sitter strain, and screening the F1 for rover behaving larvae that carried a shared recessive lethal tag (Pereira *et al*. 1995). In the present paper we show that *Csr*, which has been refractory to localization for many years, is a heterochromatic gene. Initial attempts to identify *Csr* by mapping the recessive pupal lethal tag occurred in the late 1980s and early 1990s. At that time, tools for genetic mapping were limited to large deletion sets for the X, 2nd and 3rd chromosomes and the Drosophila genome had yet to be sequenced. Furthermore, the tools available for genetic mapping in heterochromatin were in their infancy and even further limited, so mapping what is now known to be a heterochromatic gene, was, understandably, unsuccessful. In the end, it took a good understanding of the properties of heterochromatin to successfully map *Csr*.

The term heterochromatin was first introduced by Heitz (1928) to describe darkly staining regions of chromosomes that remain compact during the cell cycle; this is compared with euchromatin, which cycles through condensed and uncondensed states during the cell cycle. Heterochromatin was once considered to only silence gene expression due to the phenomenon of position effect variegation (PEV). First discovered by Muller (1930) , PEV is an effect whereby the expression of euchromatic genes is suppressed in a mosaic fashion when moved by chromosome rearrangement into, or near, heterochromatin. At the time of Muller, no genes were known to reside in heterochromatin, supporting the classification of heterochromatin as silencing chromatin. However, mapping projects (Hilliker and Holm 1975; Hilliker 1976; Marchant and Holm 1988a, 1988b; Schulze *et al*. 2001; Coulthard *et al*. 2010; Syrzycka *et al*. 2019) and the sequencing of Drosophila heterochromatin (Hoskins *et al*. 2007 ) showed that there were genes located in heterochromatin, some of which were essential to development, albeit in very low number compared with the gene density in euchromatin. As heterochromatic genes were identified, studies found that heterochromatic genes variegate when placed in euchromatic environments, showing that these genes require a heterochromatic environment for proper expression and regulation (Wakimoto and Hearn 1990, Eberl *et al*. 1993, Howe *et al*. 1995). As such, PEV is not a property limited to heterochromatin. PEV is a more general phenomenon whereby a gene moved from its native chromatin environment and into, or closely adjacent to, another chromatin environment will be susceptible to position effects.

In this study we resumed the localization of *Csr* using genetic mapping and molecular tools that were not available when originally attempting to map *Csr*. Our mapping efforts resumed in euchromatin, in the candidate region described by Pereira *et al*. (1995), but we ultimately showed that *Csr* mapped to proximal heterochromatin on the right arm of chromosome 3. We showed through a combination of deletion mapping, qRT-PCR and feeding of ecdysone hormone to larvae during development, that the alleles of *Csr* were consistent with variegating alleles of the previously characterized gene, *spookier* (*spok*). The *spok* gene is one of the Halloween group of genes essential for the production of the molting hormone ecdysone. s*pok* was first discovered by Ono *et al*. (2006), who were able to rescue lethal alleles of *spok* by feeding larvae ecdysone hormone. We showed that there is a significant difference in expression levels of *spok* between the naturally occurring rover and sitter *for* alleles. The naturally occurring rover variant of the *for* gene, and the rover-like *Csr* alleles, have higher overall levels of expression of *spok* compared with the naturally occurring sitter alleles of *for*.

## Methods and materials

### Fly strains and crosses

Flies were raised on standard yeast-sugar-agar fly medium (Burns *et al*. 2012) with stocks maintained at 25 ± 1°C and a 12 h light/dark cycle with lights on at 0600 h, as described in Anreiter *et al*. (2016). Table 1 lists all lines used in this study.

**Table 1.** *D. melanogaster* strains used in this study.

Fly strain(s)	Description	Source
*for^R^* and *for^s^*	Rover and sitter strains of the *foraging* (*for*) gene, respectively, used by Pereira *et al*. (1995) in the discovery of *Csr*.	Sokolowski lab stocks; Pereira *et al*. (1995)
*Csr* ^1^, *Csr*^2^, and *Csr*^3^	The original mutant *Csr* lines created from sitter (*for^s^*) that exhibit rover-like larval foraging behavior. The *Csr*^1^ line was lost part way through the study	Sokolowski lab stocks; Pereira *et al*. (1995)
*Csr* ^RV1^, *Csr*^RV2^, and *Csr*^RV5^	Three mutant derivatives of *Csr^3^* that exhibit sitter-like larval foraging behavior.	Sokolowski lab stocks; Pereira *et al*. (1995)
*ry* ^+5^	Wild-type stock.	Hilliker lab stocks
*Df(2Lh)C'*	Deficiency for the entire heterochromatic region on the left arm of chromosome 2 (2Lh).	Hilliker lab stocks; Hilliker and Holm (1975) and Dimirti (1991)
*Df(2Rh)M41A10*	Deficiency for entire heterochromatic region on the right arm of chromosome 2 (2Rh).	Hilliker lab stocks; Hilliker and Holm (1975) and Dimirti (1991)
*Df(3Lh)TTT*	Deficiency for a large portion of the heterochromatic region on the left arm of chromosome 3 (3Lh).	Bloomington Stock Center; Syrzycka *et al*. (2019) and Li *et al*. (2009)
*Df(3Rh)e-204* *Df(3Rh)e-1676*, *Df(3Rh)8740#18 Df(3Rh)PARP1-1*	Heterochromatic deficiencies in the right arm of chromosome 3 (3Rh; see Fig. 3)	Bloomington Stock Center; *Df(3Rh)e-204* (Koundkjian *et al*. 2004); all others in FlyBase (Öztürk-Çolak *et al*. (2024), Fitzpatrick (2005), Honda (2019), Cook (2019)
*Su(var)2-5*; Su(var)2-8^01^	Second chromosome suppressors of euchromatic position effect variegation.	From Gunter Reuter, Martin Luther University, Germany
*E(var)7^01^*; *E(var)8^48g^*	Second chromosome enhancers of euchromatic position effect variegation.	Bloomington Stock Center
*Df(3R)crb*87-5 *Df(3R)crb*87-4 *Df(3R)w5* *Df(3R)Exel6198* *Df(3R)Exel8178* *Df(3R)Exel6199* *Df(3R)F89-4*	Deficiencies which extend into the *jar*-*crb* region (cytological band 95F).	Bloomington Stock Center; Exel deficiencies are from the Exelixis collection (Parks *et al*. 2004)
*crb^S010409^*; *crb^07207^*; *crb^j1b5^*; *crb^EY06985^*	Lethal alleles of the gene *crumbs* (*crb*).	Bloomington Stock Center
*crb^COS-P1^*	A second chromosome insertion of a 33 kb clone spanning −6 kb to +26 kb from the transcription start site of *crb*.	Tepass *et al*. (1990)
*jar^2095^*; *jar^1646^*	Lethal alleles of the gene *jaguar* (*jar*).	Bloomington Stock Center

A brief description of each line is provided, along with the source of each stock.

### Complementation analysis

Complementation analysis was performed by crossing 5–10 males from 1 mutant line to 5–10 females of a second line in a vial containing 5 mL of standard fly medium, with all adults being removed after 3 days. Crosses were performed at both 25°C and 29°C when scoring for *Csr*-related recessive phenotypes or when deletion mapping, otherwise crosses were performed at 25°C. A minimum of 50 progeny were counted from each cross. A cross was considered lethal if the number of transheterozygous progeny was <5% of the total progeny and semi-lethal when transheterozygotes made up more than 5% of the total progeny but significantly less than the expected progeny had the cross been fully viable. Significance was determined by chi-square analysis with *P* < 0.05. All crosses were scored to exhaustion.

### Scoring pupal lethality

Genetic crosses used for testing pupal lethality were set up with 25 males and 25 females in a standard glass-rearing vial with 5 mL of standard fly media, and all adults removed from the vial after 3 days. To score each cross to exhaustion and verify instances of pupal death, all pupal cases in a vial were marked on day 14. On day 28, all marked pupal cases were scored as empty (having had an adult successfully eclose) or not being empty. Nonempty pupal cases on day 28 were considered pupal deaths.

### Cytology

Salivary gland polytene chromosomes from wandering third larval instar of *Csr^2^, Csr^3^*, *Csr^RV1^*, and *Csr^RV5^* were examined for chromosome aberrations. To assist in the identification of aberrations, each line was first crossed to wild-type *ry*^+5^ so chromosomes could be observed when paired with a wild-type chromosome. Chromosome banding patterns were compared with polytene banding maps available in Lindsley and Zimm (1992). To prepare a chromosome squash, individual larvae were placed in a drop of 45% acetic acid and the salivary glands removed into the acid. The salivary glands were transferred to a clean slide and covered with a drop of natural orecin dye and a cover slip. The cover slip was gently tapped with the blunt end of dissecting tweezers for approximately 10–15 s to spread out the nuclei and chromosomes in the salivary glands. Chromosomes were fixed in place after tapping by applying direct pressure to the cover slip for several seconds and sealing the cover slip to the microscope slide using clear nail polish. All aberration breakpoints were confirmed in multiple nuclei from multiple larvae.

### Modifiers of position effect variegation and lethality

Four second chromosome modifiers of position effect variegation [mod(PEV)] were placed in the genetic background of 3 *Csr*^RV^ lines (*Csr*^RV1^, *Csr*^RV2^, and *Csr*^RV5^). Two of the mod(PEV) were suppressors of variegation, Su(var)2-5 and Su(var)2-8^01^, and 2 were enhancers of variegation, E(var)7^01^ and E(var)8^48g^. *Csr^RV^* lines with a mod(PEV) were crossed to *Csr^RV^* lines without a mod(PEV) gene and the percentage of trans-heterozygous *Csr^RV^* adults emerging from the cross was compared with trans-heterozygous adults emerging from control crosses between *Csr^RV^* lines in the absence of a mod(PEV).

### Collection of Larvae

Larvae were collected as in Anreiter *et al*. (2016). Briefly, several hundred flies, per line, were placed in a standard fly bottle and capped with a collection plate (35 × 100 mm culture dish lid). Collection plates were filled with grape juice agar and sprinkled with active yeast to promote egg laying. Small holes were punctured in the side of the bottle to provide air. Flies were left overnight to clear females of fertilized eggs, after which a fresh collection plate was used to cap the collection bottle. Flies were allowed to lay eggs for 4 h, in the dark, after which the adults were removed. Collection bottles were left at 25°C, and approximately 24 h later, the collection plates were cleared of all larvae. Within 4 h of clearing, 100 newly hatched larvae were picked from the collection plate and transferred to a 100 × 15 mm petri dish filled with 35 mL of standard fly medium.

### Larval foraging testing

The foraging pathlengths of 96 h old (±2 h) foraging stage third instar larvae, collected as described above, were tested as in Anreiter *et al*. (2016). Larvae were placed in the wells of test plates containing a thin yeast-water paste, with 1 larva per well. Ten larvae were tested at the same time, corresponding to the number of wells on each test plate. As the larvae fed on the yeast and moved, they left visible paths in the yeast paste. After 5 min the path length of each larva was recorded (*n* = 40 larvae per line). Statistical differences between mean path lengths per line were determined by an ANOVA test and a Student-Newman-Keuls (SNK) a *posteriori* analysis (*P* < 0.05).

### Quantitative reverse transcription PCR

Quantitative reverse transcription PCR (qRT-PCR) was conducted on 96 h old (±2 h) third instar foraging stage larvae from the *for^R^*, *for^s^*, and *Csr^2^* lines. Third instar foraging stage larvae, 96 h old (±2 h) were collected as described above for a larval foraging test. Each qRT-PCR test used 10 larvae and was replicated 5 times per line. Reverse transcriptase—polymerase chain reactions were completed using an iTaq Universal SYBR Green One-Step Kit and a Rotor-Gene Q machine used as both a thermocycler and spectrophotometer. Expression levels of the gene of interest were compared relative to expression of a β-Actin control, and statistical significance was determined by a 2-tailed *t*-test. See Table 2 for a list of primers used.

**Table 2.** Primer pairs for qRT-PCR and for sequencing the exonic regions of the *D. melanogaster* spok, PARP1, and alg-2 genes.

Gene	Forward primer	Reverse primer
*spok—*genomic sequencing	TGTTGAGAACTATTTACTAAGGCTGAA	TGCACGAGAATTTGCTGCTA
	TGCGTACAAGAGAGCTGCTG	AAAATGGTACCAGCCAAGGT
	CGGGATATCCATGTGACGTA	AAAAACCGCTCAGGATCAAA
	TGAGCGAAAGCTTTTGGAAT	GCATTCGGCGTTATGTGACT
*spok—*qRT-PCR	CGGTGATCGAAACAACTCAC	TGTCGCCGAGCTAAATTTCT
*PARP1—*genomic sequencing	TCCAAATGACCACCCTTTCT	CTGTCGTTCCCGCTAGTTTC
	TGAAACGGTTACACCAAACG	GAAGGGCATCCCCAACTAAG
	GCACTTCCAGCGTTATGACA	CCAATACGGTTACGACCACA
	TATCGCCCACGGTCATTATT	ATGCCAAAAACGTGTTGGTT
	CGGGATAGTCGACTTGCATT	GCTTCTCCTTGGCGAGATAC
	GATTTGCGACAAATGCTTGA	AAGGAATTTCAACCCCATCA
	GGCACGGATCACGTTTAACT	GTCATTTAAGCCCGCTGTGT
*PARP1—*qRT-PCR	CAAGAACTGGACGAGCCACT	TTGGCATCATGAAATGCAGA
*alg-2—*genomic sequencing	AACTGTTGTCTGTGCGAATAGTCT	ACCCGAAACGCCATACAAT
	ACTTTAGGCAGTGGCAGCTT	CTGGTCTCTGGTTTTGCTCA
*alg-2—*qRT-PCR	CTTTGGGCGTGGTACAATTC	TGATCCCATCCAAGTCAGTG
β-Actin- qRT-PCR	GTTGGAGAAGTCCTACGAGCTG	ATGGAGTTGTAGGTGGTCTCGT

For each gene, genomic sequencing primers are listed from top to bottom to correspond to the 5′ end to the 3′ end of the gene, respectively.

### 20-hydroxyecdysone (20E) feeding experiments

The hormone 20E was fed continuously to larvae during development, as in Ono *et al*. 2006, except 20E was mixed into standard fly media instead of in a live yeast paste. To lace standard fly media, 20E was dissolved in 3% DMSO before adding it to the food. Appropriate amounts of 20E in DMSO were added to the food to produce final 20E concentrations ranging from 5 to 100 μM. Unlaced food for controls was prepared in the same manner, with 3% DMSO added to the food but with no 20E present. First-instar *for^R^* and *for^s^* larvae, all within 4 h post hatch, were collected as above for larval foraging tests, and placed in glass vials containing 5 mL of fly media containing 20E with the following concentrations in μM: 0, 5, 10, 15, 20, 25, 30, 35, 40, 45, 50, 100. Fifty first instar larvae per strain were then added to each vial and placed in standard rearing conditions at 25°C. Vials were checked every 12 h over 28 days to count and/or score the number of larvae, pupae and adults in each vial, as above, with all pupal cases in a vial being marked on day 14 to score for pupal lethality.

### Sequencing genomic DNA

Genomic DNA was isolated by grinding up a single male in a 1.5 mL polypropylene Eppendorf tube containing 46 μL of lysis buffer. Lysis buffer consisted of 10 mM Tris (pH 8.2), 1 mM EDTA, and 25 mM NaCl. After grinding, 4 μL of 20 mg/mL Proteinase K was added to the tube and incubated at 37°C for 30 min and then incubated at 95°C for 10 min to deactivate the Proteinase K. One μL of the fly homogenate was used for each standard 20 μL PCR reaction, using primers listed in Table 2. Amplified DNA was purified using a QIAquick PCR purification kit, available from Qiagen, and Sanger sequencing completed at the facilities of Bio Basic Inc., 20 Konrad Crescent, Markham, Ontario.

## Results

### Searching for *Csr* in the euchromatic arms of chromosome 3

#### Deletion mapping in the original Csr candidate region from Pereira *et al*. (1995)

Pereira *et al*. (1995) mapped the pupal lethality tag to a region bounded by 2 deletions in third chromosome euchromatin, *Df(3R)crb*87-5 and *Df(3R)crb*87-4. Our deletion analysis included those original 2 deletion lines and an additional 4 deletions: *Df(3R)*w5, *Df(3R)*6198, *Df(3R)*8178, and *Df(3R)*6199. The 6 deletions were tested in all *inter se* combinations to produce the deletion map found in Supplementary Fig. 1 (data shown in Supplementary Tables 1 and 2), and then the deletions were tested for complementation with alleles of *jar*, *crb*, and the *Csr^RV^* lines at 29°C (data shown in Supplementary Tables 3 and 4). Our deletion analysis effectively narrowed down the candidate region for the pupal lethal tag of *Csr* to an area containing 9 predicted or known coding regions between the genes *jaguar* (*jar*) and *crumbs* (*crb*), inclusively.

### New recessive phenotype tags discovered

While mapping in the *jar-crb* region, all adult progeny were carefully screened for additional phenotype tags and several new recessive phenotypes in the *Csr* and *Csr^RV^* lines were discovered. The severity and frequency of every new phenotype tag increased with increases in temperature (25°C compared with 29°C). These new tags included wing phenotypes (wrinkling and vein abnormalities), abdominal banding abnormalities, and low-level pupal lethality at 25°C.

#### Low level pupal lethality at 25°C in Csr^1^, Csr^2^, and Csr^3^ lines

The *Csr^1^*, *Csr^2^*, and *Csr^3^* lines displayed low-level pupal lethality at 25°C. This pupal lethality at 25°C was newly discovered as the mapping efforts of Pereira *et al*. (1995) focused on pupal lethality at 29°C. This low-level pupal lethality at 25°C occurred at the pharate stage and appeared in <5% of the total progeny, and was more severe at 29°C.

#### Wrinkled wings and wing vein abnormalities

Two newly observed wing phenotypes were discovered: wrinkled wing and wing vein abnormalities that included extra or branching veins. Changes to wing veins were more common than wrinkled wings and the vein phenotypes were quite varied. Drawings of various vein phenotypes are provided in Supplementary Fig. 2. The 2 wing phenotypes were observed in all mapping crosses in the *jar-crb* region, to varying degrees, but neither wing phenotype was observed in the original homozygous *Csr* lines.

#### Abdominal banding abnormalities

**Fig. 1.**
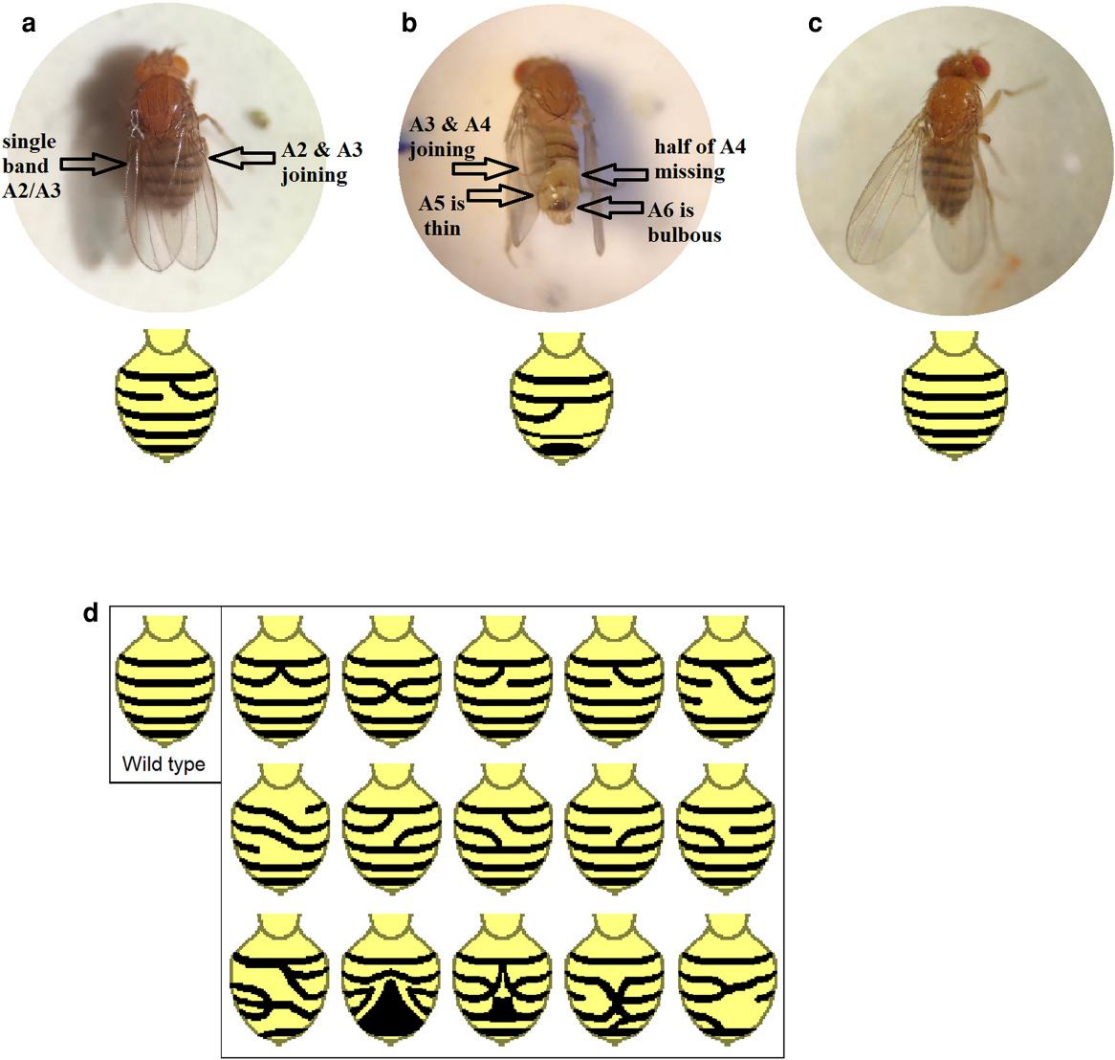
A new recessive phenotype tag in the *Csr^RV^* lines produces abdominal banding abnormalities. Examples of variability in *D. melanogaster* abdominal banding abnormalities in female progeny of crosses involving the *Csr^RV2^* line (a and b) and wild-type c) are shown. a): minor abnormality with one side of bands A2 and A3 coming together at the dorsal midline with only a single band on the side opposite. b): a more severe abnormality with one half of band A4 missing and the other half of A4 curving upwards to meet band A3 at the dorsal midline. Band A5 is thin and band A6 is bulbous-looking. c): wild-type banding in a *for^s^* female. d): representative drawings of additional banding patterns observed with crosses involving the *Csr^RV2^* line during the initial investigation of the phenotype (with wild-type for comparison on the left hand side).

An abdominal banding phenotype was observed in 1–2% of adult homozygotes in the original *Csr* lines and the *Csr^RV^* lines (see Fig. 1). The abdominal banding phenotype was also present in trans-heterozygous combination of original *Csr* chromosomes with *Csr^RV^* chromosomes. The majority of abdominal banding abnormalities were limited to 1 or 2 adjacent sections of the abdomen with 1 or 2 abdominal bands curving up, or down, to join the adjacent band at the dorsal midline. The other halves of the bands either extended normally to the midline or were missing altogether. More severe banding abnormalities were observed in trans-heterozygotes combinations involving the *Csr^RV1^* and *Csr^RV2^* chromosomes. In these severe cases, 3 or more bands curved to form complex banding patterns, or 1 band curved and joined a band 2 segments away, disrupting the band in between. In *Csr^2^* and *Csr^3^*, the abdominal abnormality typically appeared in <5% of adult progeny when raised at 25°C, but appeared in between 5 and 10% of progeny at 29°C. Trans-heterozygote combinations of *Csr^RV^* lines also exhibited banding abnormalities at similar percentages at 25°C. Due to the temperature-sensitive pupal lethality present on the *Csr^RV^* chromosomes, an accurate frequency of abdominal banding defects could not be determined for trans-heterozygote combinations of *Csr^RV^* chromosomes.

### Abandoning the jar-crb region as a candidate region for Csr

The abdominal banding abnormalities observed in *Csr* were observed in a large number of crosses involving *crb* mutants, and *crb* in combination with *Csr*. Additionally, the new wing phenotypes were also found with *jar* mutants in combination with *Csr* lines, making *jar* and *crb* potential candidates for *Csr*. However, additional analyses were unable to find any evidence to map *Csr* to *jar* and *crb*, or any of the 7 predicted coding region between them. For example, the 9 coding regions between *jar* and *crb*, inclusive, were sequenced in *Csr^2^*, *Csr^3^*, *Csr*^RV1^, *Csr*^RV2^, and *Csr*^RV5^ and no nucleotide differences were found in any line when compared with each other or when compared with the *for^s^* genetic background control. We also used the cosmid line, *crb^COS-P1^*, to investigate *crb* as a potential *Csr* or lethal tag candidate. *crb^COS-P1^* contains a crb plasmid that rescues lethality in lethal crb mutants (Tepass *et al*. 1990). We placed *crb^COS-P1^* in a *Csr^RV2^* background and then crossed the new line to 2 *crb* mutants, *crb^EY06985^* and *crb^S010409^*. Crosses produced abdominal banding abnormalities in a large percentage of heterozygous progeny in crosses without *crb^COS-P1^*, while in the presence of the *crb^COS-P1^*, the abdominal banding abnormalities were completely rescued (Supplementary Table 5). However, *crb^COS-P1^* was unable to rescue pupal lethality (Supplementary Table 6).

The pupal lethal tag for Csr was only uncovered with deletions that were deficient for both *jar* and *crb*. Consequently, we abandoned the *jar*-*crb* region. We hypothesized that the function of the gene associated with Csr was more likely to overlap with the functions of *jar* and *crb* to produce the abdominal and wing phenotypes, rather than directly mapping to this region.

### Searching for a new candidate region for *Csr*

Our deletion analysis was expanded to include the remaining euchromatic regions of the third chromosome. This new deletion analysis failed to produce any additional candidates for the temperature sensitive lethal tag and only uncovered several secondary site lethals shared between single mapping deletions and single *Csr^RV^* chromosomes. As such, we returned to the original analysis of Pereira *et al*. (1995) and found a critical result that allowed us to map *Csr*. All polytene chromosomes analyzed by Pereira *et al*. (1995) had at least 1 heterochromatic break, suggesting that the normal expression of *Csr* might be experiencing modification by the presence of heterochromatin, or *Csr* could be associated with heterochromatin instead of euchromatin.

### Newly analyzed *Csr* lines all contain heterochromatic breaks in polytene chromosome squashes

We analyzed the polytene salivary gland chromosomes in 4 lines that were not previously analyzed by Pereira *et al*. (1995): *Csr^2^*, *Csr^3^*, *Csr*^RV1^, and *Csr*^RV5^. As in Pereira *et al*. (1995), all newly analyzed *Csr* lines had chromosome rearrangements with at least 1 heterochromatic break.

**Fig. 2.**
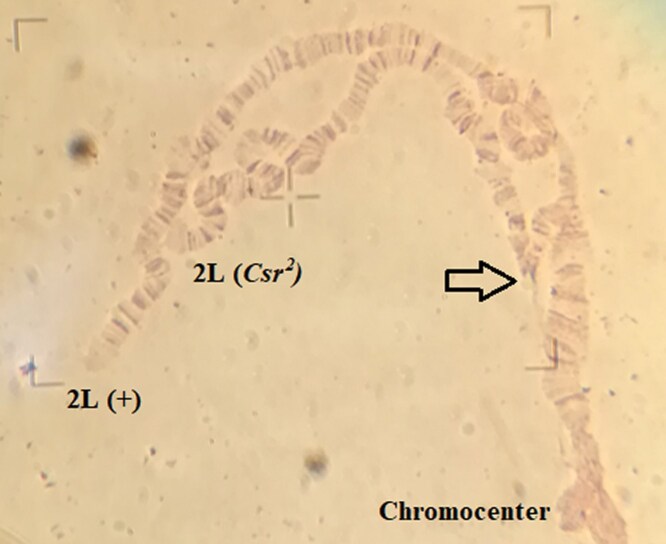
*D. melanogaster* salivary gland polytene squash from a *Csr^2^*/*ry*  ^*+*^  ^*5*^ heterozygous larvae reveals the presence of a heterochromatic break in the *Csr^2^* line. A heterochromatic insertion at band 36B, shown by the arrow, is visible on the left arm of chromosome 2 (2L) in this polytene squash from a *Csr^2^/ry*  ^*+*^  ^*5*^ heterozygous larvae. It is a typical observation with heterochromatic insertions into euchromatin to see the ends of paired chromosomes unzip distal to the heterochromatic insertion during slide preparation, as seen here. The thin line extending from 36B on the *Csr^2^* chromosome arm is heterochromatin and leads back to the chromocenter at the bottom right of the slide. Although it is difficult to distinguish thin heterochromatic strands from ectopic pairing in individual nuclei, the features described here, including the thin line extending from 36B, were consistent in multiple nuclei from independent squashes.

A salivary chromosome squash for a *Csr^2^*/*ry^+5^* heterozygote is shown in Fig. 2. Analysis showed that *Csr^2^* had a heterochromatic insertion at 36B on 2L, giving a new order of 21-36B|het|36B-40, where “het” is short for heterochromatin. There were no other visible aberrations on any other chromosome arms in *Csr^2^*. *Csr^3^* had an inversion loop in 3L with 1 break at 74C and the other break in heterochromatin, giving a new order of 61-74C|het| 80-74C | het|82–100. It was not possible with polytene chromosome squashes to determine whether *Csr^3^* had a pericentric inversion, or an inversion limited to 3L. Genetic evidence in the analysis of mod(PEV) (reported below), points to a pericentric inversion with the new chromosome order placing *Csr* and the lethal tag in close proximity to euchromatin.

Salivary chromosome squashes for the 2 revertant lines, *Csr*^RV1^ and *Csr*^RV5^ included the same aberrations observed in *Csr*^3^, but each revertant had additional aberrations involving heterochromatin. *Csr*^RV1^ had additional breaks at 75C and heterochromatin, giving a new order of |61-74C|het|80-75C|het|74C-75C|het|82-100. *Csr*^RV5^ had additional breakpoints at 87B and heterochromatin, giving a new order of |61-74C|het|87B-82|het|80-74C|het|87B-100.

With heterochromatin breaks present in all the lines we tested, and in all the lines analyzed by Pereira *et al*. (1995), these results demonstrated that heterochromatin was likely involved in some manner with all the *Csr* and *Csr^RV^* lines. Consequently, we hypothesized that the phenotypes associated with *Csr* and/or the lethal tag could be a result of PEV brought on by chromosome rearrangements bringing heterochromatin and euchromatin in close juxtaposition.

### Modifiers of PEV showed that the pupal lethal tag of *Csr* was linked to a variegating heterochromatic gene

To test for PEV, 4 modifiers of mod(PEV) were placed in the genetic background of 3 *Csr*^RV^ lines (*Csr*^RV1^, *Csr*^RV2^, and *Csr*^RV5^). Two of the modifiers were suppressors of variegation, *Su(var)2-5* and *Su(var)2-8^01^*, and 2 were enhancers of variegation, *E(var)7^01^* and *E(var)8^48g^*. The percentage of *Csr*^RV^ trans-heterozygous adults emerging from crosses between *Csr*^RV^ line with, or without, a mod(PEV) was scored and compared in Table 3.

**Table 3.** The presence of *D. melanogaster* mod(PEV)s modifies the total number of adults recovered in various transheterozygous combinations of Csr^RV^ lines.

	With mod(PEV)		Without mod(PEV)		
Combination tested at 29°C	Total progeny	Total Csr^RV#^/Csr^RV#^	Total progeny	Total Csr^RV#^/Csr^RV#^	*P* (*Χ*^2^)
Su(var)2-5 with					
*Csr^RV1^*/*Csr^RV2^*	271	12	309	22	0.134
*Csr^RV1^*/*Csr^RV5^*	156	1	136	2	0.396
*Csr^RV2^*/*Csr^RV5^*	148	11	168	25	0.038*
Su(var)2-2-801 with					
*Csr^RV1^*/*Csr^RV2^*	147	12	122	20	0.031*
*Csr^RV1^*/*Csr^RV5^*	164	0	189	13	0.0008*
*Csr^RV2^*/*Csr^RV5^*	67	12	87	12	0.109
E(var)7^01^ with					
*Csr^RV1^*/*Csr^RV2^*	116	3	159	11	0.085
*Csr^RV1^*/*Csr^RV5^*	102	2	120	2	0.791
*Csr^RV2^*/*Csr^RV5^*	33	4	59	13	0.345
E(var)8^48g^ with					
*Csr^RV1^*/*Csr^RV5^*	142	16	167	9	0.0004*
*Csr^RV2^*/*Csr^RV5^*	108	41	132	34	<0.0001*

Crosses with a significant difference (*P* < 0.05) are marked with an asterisk. Suppressors of variegation decrease the number of transheterozygous Csr^RV^ adults recovered while enhancers of variegation had the opposite effect. The direction of the effect is consistent with a variegating heterochromatic gene.

In total there were 11 combinations of *Csr*^RV^ lines and mod(PEV) genes tested (the 12th combination, E(var)8^48g^; *Csr*^RV1^/*Csr*^RV2^ failed to produce any progeny after repeated attempts and was abandoned). As shown in Table 3, 5 chromosome combinations showed significant differences in lethality in the presence of a mod(PEV). Three of the five significant cases showed an increase in pupal lethality in the presence of a Su(var), while 2 cases showed a decrease in pupal lethality in the presence of an E(var). The opposing effects of the Su(var)s and E(var)s on pupal lethality indicated that this phenotype was exhibiting PEV. Su(var)s and E(var)s are named after their effect on variegating euchromatic genes, suppressing and enhancing variegation, respectively. Our results showed the modifiers producing the opposite effects with enhancement of variegation by Su(var)s and suppression of variegation by E(var)s. Such an opposite effect is indicative of a variegating heterochromatic gene, leading to the hypothesis that the lethal tag phenotype was a result of a variegating heterochromatic gene.

### Searching for *Csr* in proximal autosomal heterochromatin

#### Deletion mapping in heterochromatin

Deletion mapping in heterochromatin began with large deletions of the proximal autosomal blocks of heterochromatin: *Df(2Lh)C*’ and Df(2Rh)*M41A10* were used for mapping second chromosome proximal heterochromatin; *Df(3Lh)TTT* and Df(3Rh)8740#18 were used for mapping the third chromosome proximal heterochromatin. *Csr*^2^, *Csr^3^*, *Csr*^RV1^, and *Csr*^RV2^ were each crossed to the 4 large heterochromatic deletions at 29°C. Two phenotype tags related to *Csr* were scored in the deletion mapping experiments: temperature sensitive pupal lethality and abdominal banding abnormalities. Of the 4 heterochromatic deletions, only Df(3Rh)8740#18, in combination with *Csr and Csr*^RV^ alleles, produced progeny with abnormal abdominal banding and pupal lethality at 29°C. As such, a new candidate region emerged for *Csr* and its lethal tag, located in the proximal heterochromatin on the right arm of chromosome 3 (hereafter referred to as 3Rh).

### Searching for *Csr* in the proximal heterochromatin of the right arm of chromosome 3 (3Rh)

**Fig. 3.**
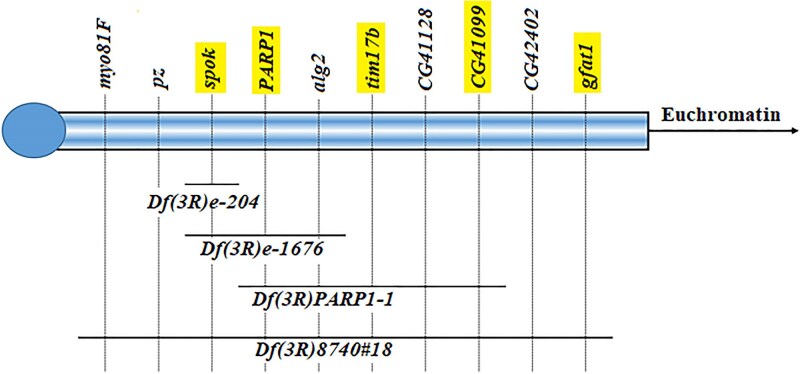
Deletion analysis in 3Rh narrowed the candidate region for *Csr* to 3 genes: spok, *PARP1* and *alg-*2. Known or predicted coding regions in 3Rh are shown as vertical lines. The distance between genes is not to scale and genes known to be essential are highlighted. Deletions are represented by horizontal lines and the centromere by a circle. Both the pupal lethal tag and the abdominal banding abnormalities were localized to 3Rh using *Df(3Rh)8740#18*, with their location narrowed down to the region spanning from *spok* to *alg-2* based on a failure to complement *Df(3Rh)e-1676*. Only the pupal lethal tag was uncovered by *Df(3R)PARP1-1*, localizing the pupal lethal tag to the essential gene *PARP1*. Abdominal banding abnormalities were uncovered by *Df(3Rh)e-204*, mapping the abdominal banding phenotype to *spok*.

Three smaller deficiencies in 3Rh, *Df(3Rh)e-204*, *Df(3Rh)e-1676*, and *Df(3Rh)PARP1-*1, were used to narrow down the candidate region in 3Rh. These 3 deletions were previously characterized molecularly (Table 1) and shown on the map in Fig. 3. Of the three 3Rh deficiencies tested, *Df(3Rh)e-1676* uncovered both the temperature sensitive pupal lethal phenotype and the abnormal abdominal banding phenotype in combination with *Csr^2^*, *Csr^RV1^*, and *Csr^RV2^* (Table 4), narrowing down the candidate region for *Csr* to 2 essential heterochromatic genes, *spok* and *PARP1*. The deletion mapping successfully separated, genetically, the pupal lethal tag phenotype and abdominal banding phenotype as follows: *Df(3R)PARP1-1* uncovered the temperature sensitive pupal lethal tag but not the abdominal banding phenotype, mapping the pupal lethal tag to the previously characterized essential gene *PARP1* (Tulin *et al*. 2002); *Df(3Rh)e-204* uncovered the abdominal banding phenotype along with a low level pupal lethality consistent with the *Csr^2^* and *Csr^3^* lines, mapping the abdominal banding phenotype to *spok*. Additional crosses using the null *spok* allele, *spok*^z-712^, confirmed the abdominal banding abnormalities were mapping to *spok*, along with a low-level pupal lethality that was much less severe than the pupal lethality associated with *PARP1*.

**Table 4.** *D. melanogaster* crosses between Csr or Csr^RV^ alleles and 3Rh deletions and the spok^z-712^ null at 29°C.

Deletion/Mutant	*Csr* or *Csr^RV^* allele	TM3 progeny	Transheterozygous TM3^+^ progeny	# of pupal deaths	# of abdominal banding defects
*Df(3R)8740#18*/TM3	*Csr*-2	82	72	12	11
*Df(3R)8740#18*/TM3	*Csr^rv1^*/TM3	54	67	13	5
*Df(3R)8740#18*/TM3	*Csr^rv2^*/TM3	41	36	5	3
*Df(3R)e-1676*/TM3	*Csr*-2	69	80	5	4
*Df(3R)e-1676*/TM3	*Csr^rv1^*/TM3	72	41	11	3
*Df(3R)e-1676*/TM3	*Csr^rv2^*/TM3	41	32	6	3
*Df(3R)PARP1-1*/TM3	*Csr*-2	43	44	4	0
*Df(3R)PARP1-1*/TM3	*Csr^rv1^*/TM3	53	33	9	0
*Df(3R)PARP1-1*/TM3	*Csr^rv2^*/TM3	56	31	23	0
*Df(3R)e-204*/TM3	*Csr*-2	53	31	1	1
*Df(3R)e-204*/TM3	*Csr^rv1^*/TM3	46	45	0	2
*Df(3R)e-204*/TM3	*Csr^rv2^*/TM3	77	34	2	14
*spok^z-712^*/TM3	*Csr*-2	69	45	3	2
*spok^z-712^*/TM3	*Csr^rv1^*/TM3	70	44	2	2
*spok^z-712^*/TM3	*Csr^rv2^*/TM3	51	29	2	5

TM3 refers to progeny that have a TM3 balancer chromosome [containing the bristle marker Stubble (Sb) and the wing marker Serrate (Ser)]. The number of adults displaying abnormal abdominal banding were scored along with the number of pupal deaths.

### Sequencing spok, PARP1 and alg-2 in Csr and Csr^RV^ lines

The coding regions of *spok*, *PARP1*, and *alg-2* were sequenced in *Csr^2^*, *Csr^3^*, *Csr*^RV1^, *Csr*^RV2^, and *Csr*^RV5^ (the nonessential gene *alg-2* was included in this, and subsequent analyses since it fell within the endpoint of *Df(3R)PARP1-1*). Table 2 lists the primers used for sequencing. There were no detectable differences between any *Csr* or *Csr^rv^* lines in the *PARP1* or *alg-2* genes when compared with the *for^s^* control genetic background. The *for^s^* control genetic background was identical in all 3 genes to the sequences established by the Drosophila heterochromatin sequencing project (Hoskins *et al*. 2015).

**Fig. 4.**
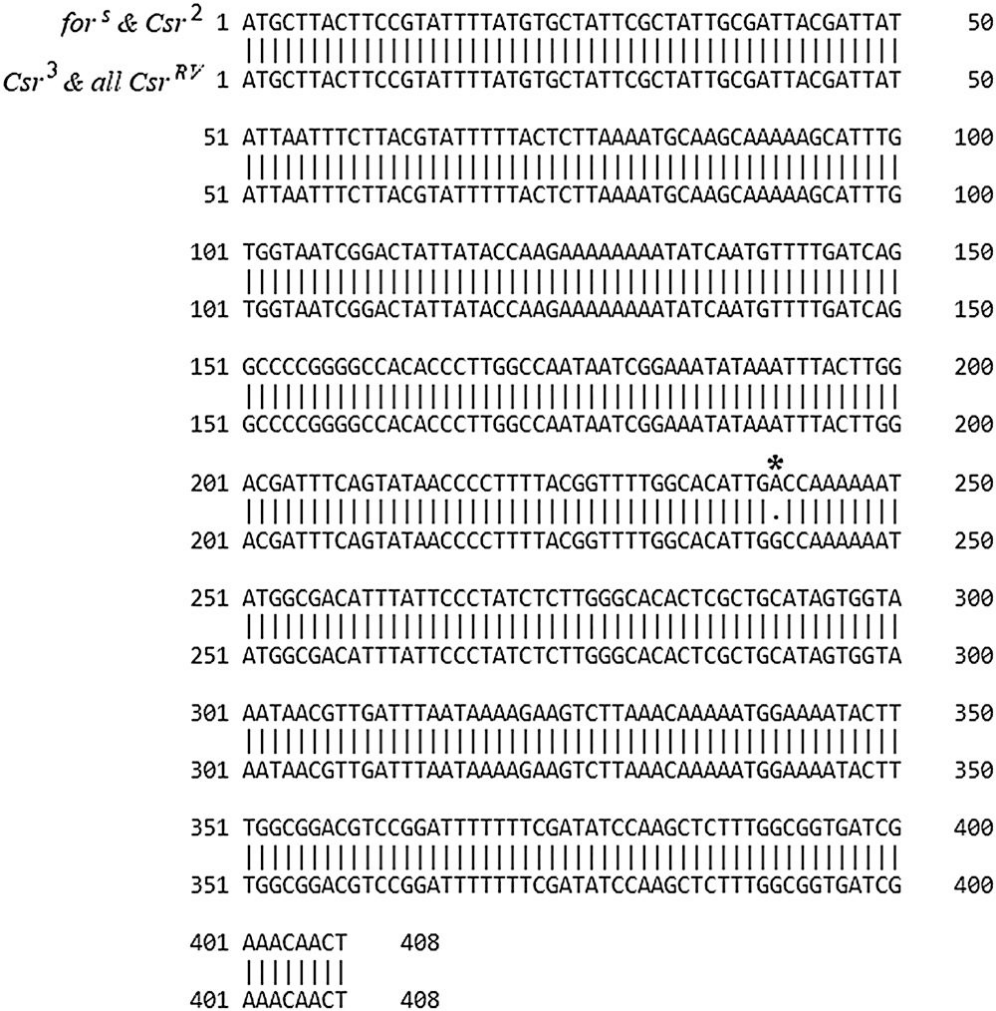
Sequence alignment of exon 1 in *D. melanogaster's spok* gene between *for^s^* and *Csr* lines, showing a single nucleotide polymorphism was present in *Csr^3^* and the *Csr^RV^* lines derived from Csr^*3*^. Sequence comparison in *spok* exon 1 between *for^s^* and multiple *Csr* lines (*Csr^2^*, *Csr^3^*, *Csr^RV1^*, *Csr^RV2^* and *Csr^RV5^*) is shown. The *Csr^2^* line contained no nucleotide differences and matched the *for^s^* genetic background. *Csr^3^* and the *Csr^RV^* lines (derived from *Csr^3^*) all share a single A to G) nucleotide polymorphism at location +241 in the first exon of *spok*.

For the *spok* gene, *Csr^2^* had no detectable differences compared with the *for^s^* control genetic background, but *Csr^3^* contained a single A to G nucleotide polymorphism in the first exon of *spok* at nucleotide +241, producing a Threonine to Alanine amino acid change (Fig. 4). This single nucleotide polymorphism was also observed in the 3 *Csr^RV^* lines sequenced, which was expected because all the *Csr^RV^* lines were derived from *Csr^3^*, but the *Csr^RV^* lines did not contain any additional nucleotide differences compared with each other or the *for^s^* control genetic background.

### Genetic and behavioral analyses confirm that *Csr alleles are* consistent with variegating alleles of the *spok* gene

#### Comparing expression levels of spok, PARP1 and alg-2 using qRT-PCR

**Fig. 5.**
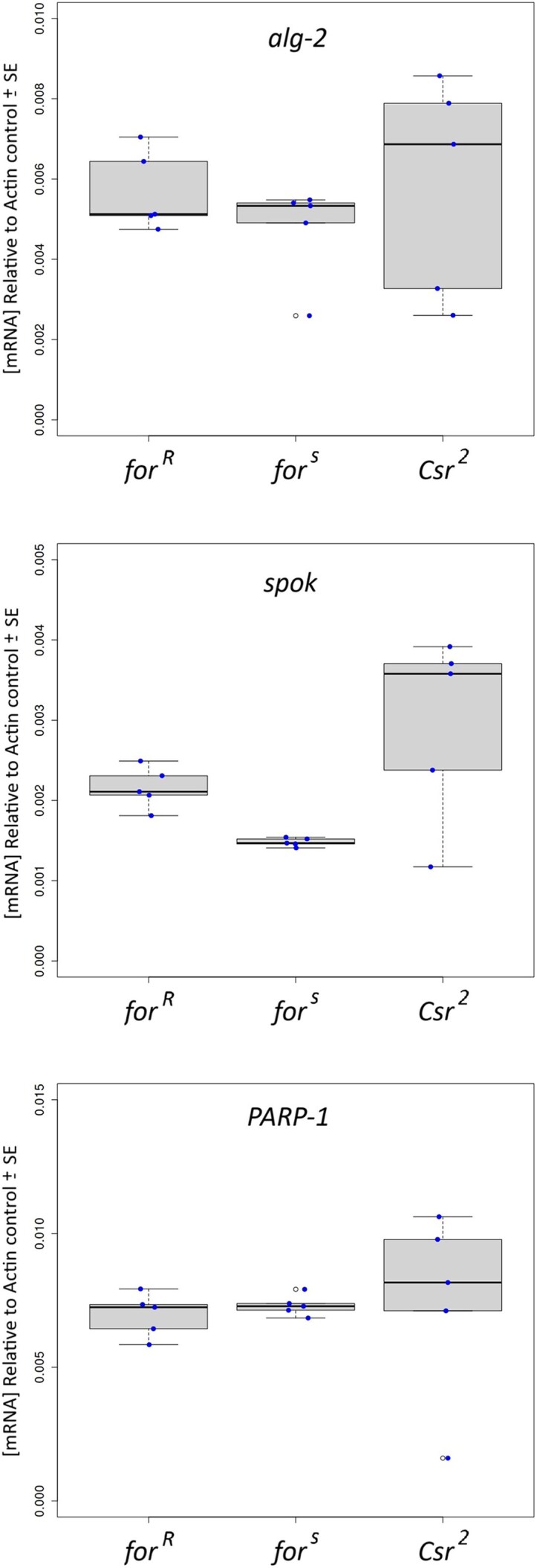
A comparison of expression levels of 3 *D. melanogaster Csr* candidate genes, using qRT-PCR, uncovered a significant difference in *spok* expression between lines with sitter behavior (*for^s^*) and rover behavior (*for^R^* and *Csr^2^*). qRT-PCR was conducted on *for^R^*, *for^s^* and *Csr^2^* third instar foraging larvae (96 h old ±2 h). Expression levels are shown as the concentration of mRNA relative to the Actin reference sample. Each qRT-PCR used 10 larvae and was replicated 5 times per line. Significant differences in expression levels between lines were only found with the *spok* gene. *for^R^* and *Csr^2^* had similar expression levels in *spok* (0.0022 ± 0.0003 and 0.0030 ± 0.0012), but both had expression levels in *spok* significantly higher than *for^s^* (0.0015 ± 0.0001).

Quantitative real-time PCR was conducted on third instar larvae of *Csr*^2^, *for^s^*, and *for^R^* to see if a candidate for *Csr* could be found based on the hypothesis that *Csr* expression may show rover/sitter differences in the same direction as *for^s^* and *for^R^*. Larvae were collected and tested in the same manner as for a larval foraging assay. Expression levels were tested for *spok*, *PARP1*, and *alg-2* (Fig. 5). No differences in expression levels were found for the *PARP1* and *alg-2* genes. For the *spok* gene, *for^s^* showed significantly lower levels of *spok* expression compared with both *Csr*^2^ and *for^R^*. These data establish a link between *spok* and the *for* gene with *spok* expression differing between *for^s^* and *for^R^*, and *Csr*^2^ expression level being *for^R^*-like such that higher *spok* expression was seen in lines exhibiting rover behavior.

#### Developmental effects of feeding varying concentrations of 20E to *for^s^* larvae

The *spok* gene is a member of the Halloween group of genes that are essential for the production of the molting hormone 20-hydroxyecdysone, also known as 20E (Rewitz *et al*. 2006). With our genetic mapping and qRT-PCR analysis showing a rover-sitter difference in third instar larvae *spok* expression, it was hypothesized that *Csr* mapped to *spok*. We tested the relationship between *spok* and *for* through a third instar larval foraging behavior assay performed with rover (*for^R^*) and sitter larvae (*for^s^*) raised on food containing 20E ecdysone hormone.

Feeding 20E to larvae was modeled after Ono *et al*. (2006) who showed that feeding 20E laced yeast paste to larvae rescued lethality in *spok* null alleles. We tested *for^R^* and *for^s^* larvae raised in solid fly medium with concentrations of 20E ranging from 0 μm as a control, to 100 μm. Table 5 shows the observations of the development of *for^s^* larvae (the genetic background of the *Csr* mutant lines) raised on 20E laced solid fly medium along with visible phenotypes observed. Results shown in Table 5 are described as follows: as the concentration of 20E increased, the time for wandering larvae to appear also increased, with higher concentrations leading to larval death. Larvae began to die, first as wanderers, and then in earlier and earlier stages as concentrations of 20E increased, until no wandering larvae appeared in the highest concentrations. In concentrations where larvae pupated, pupal development slowed, and the percentage of pupal deaths increased as the concentration of 20E increased. The stage of pupal death moved earlier as concentrations increased, with all pupae dying at the highest 20E concentrations. Under 20E concentrations where adults eclosed, 4 visible phenotypes were observed. These phenotypes were abnormal abdominal banding, an absence of wings, missing legs, and uncoordinated walking with an inability to fly.

**Table 5.** Observed effects of raising for^s^  *D. melanogaster* larvae (*n* = 50) on varying concentrations of 20E.

[20E] (μm) Observation	0	5	10	15	20	25	30	35	40	45	50	100
# days for wandering larvae to appear	3.5	3.5	3.5	4	4	4.5	5	5.5	6	6.5	X	X
Dead wanderers on side of vial	0	0	0	4	9	8	16	18	16	4	X	X
# pupae	48	42	43	45	42	24	23	11	8	X	X	X
# days for adults to begin eclosing	9	9	9	9.5	10	11	11.5	11.5	12	X	X	X
# pupal deaths	0	0	4	21	20	22	22	10	8	X	X	X
early pupal death	0	0	0	0	0	0	0	9	8	X	X	X
mid-pupal death	0	0	0	0	1	3	12	1	X	X	X	X
pharate death	0	0	4	21	16	17	10	0	X	X	X	X
eclosion deaths	0	0	0	0	3	2	0	0	X	X	X	X
Adult abdominal banding abnormalities	0	1	3	4	4	2	2	0	X	X	X	X
Wingless adults	0	0	0	1	3	0	0	0	X	X	X	X
Adults missing legs	0	0	0	0	7	1	0	0	X	X	X	X
Uncoordinated walking + can't fly	0	0	0	0	0	3	1	1	X	X	X	X

Developmental times for larvae were scored along with the number of pupal deaths and any visible adult phenotypes. Cells marked with an “**X**” were impossible to count because all animals died in an earlier stage of development.

### Sitter *(for^s^*) larvae raised in 10 μM 20E foraged like rover larvae (*for^R^)*

We attempted to change larval foraging behavior in the same direction as *Csr* (from sitter to rover) by raising larvae in 20E hormone laced food. The expectation was that feeding 20E to sitter larvae will increase the amount of available 20E for larvae as in the rescue of *spok* nulls in Ono *et al*. (2006), thereby phenocopying an increase of *spok* expression.

**Fig. 6.**
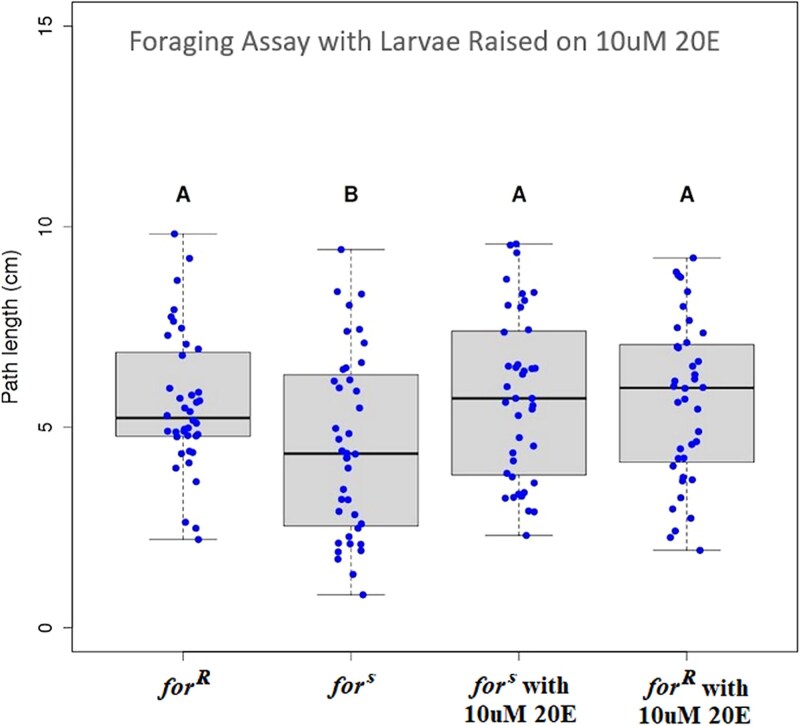
Foraging path lengths of sitter larvae (*for*^s^) are rover-like when raised in food laced with 10 μM 20-hydroxyecdysone (20E). Path lengths (cm) of *D. melanogaster* for^R^ and for^s^ control lines differed significantly (5.59 ± 0.27 cm and 4.36 cm ± 0.36 cm, respectively; *n* = 40 per line). Path lengths for for^R^ and for^s^ larvae raised on 10 μM 20E laced food (5.67 ± 0.32 cm and 5.77 ± 0.33 cm, respectively; *n* = 40 per line) were significantly longer than the for^s^ control, but not significantly different than each other or the for^R^ control. A SNK a posteriori analysis (*P* < 0.05) revealed 2 groups: group A consisted of the for^R^ rover control, for^s^ raised on 10 μM 20E and for^R^ raised on 10 μM 20E while group B consited of the for^s^ sitter control.

The concentration of 20E in the food used to raise larvae was chosen to be 10 μm for 2 reasons. First, 10 μm was the highest concentration of 20E that did not have significantly visible effects on the development of *for^s^* larvae (Table 5). It was critical to choose a feeding concentration that did not affect time to development to ensure larvae were still foraging, and not wandering, during the larval foraging assay, as wandering larval pathlengths are significantly longer than those of foraging larvae. Second, when raised on 10 μm 20E-laced food, some adults were observed with the abdominal banding pattern abnormalities we mapped to the *spok* gene. Results, visualized in Fig. 6, show that, as expected, the rover and sitter controls raised on food with no 20E differed significantly in larval path length (mean path lengths of 5.59 ± 027 cm and 4.56 ± 0.36 cm, respectively). Both 10 μM feeding tests did not differ significantly from control line *for^R^* and were both significantly longer than control line *for^s^* (mean path lengths were 5.77 ± 0.33 cm for *for^s^* larvae raised on 10 μm 20E and 5.67 ± 0.32 cm for *for^R^* larvae raised on 10 μm 20E). With *for^R^* larval path lengths fed no 20E not differing from *for^R^* larval pathlengths when fed 10 μM 20E, we confirmed that the larvae were still foraging and were not wandering due to the feeding of 20E, and by feeding 10 μM 20E to *for^s^* larvae, the behavior of the sitter control became rover-like. In combination with the mapping, genetic analyses, and qRT-PCR results, the change in behavior of *for^s^* larvae through the feeding of 20E hormone confirmed that Csr alleles were consistent with variegating alleles of *spok*.

## Discussion

Several breakthroughs in our mapping demonstrated that *Csr* mapped to a variegating heterochromatic region: heterochromatic breaks were observed in the polytene chromosomes of every *Csr* line, and modifiers of PEV modified the pupal lethal tag in a manner consistent with a variegating heterochromatic gene. Additionally, the high level of phenotypic variation observed in the recessive phenotype tags supported our finding that we were working with variegating genes. Finally, all phenotypes used in the mapping of *Csr* were enhanced by increases in temperature; developmental temperature was one of the first known mod(PEV), with increasing temperatures displaying increased suppression of euchromatic PEV (Hartmann-Goldstein 1967; Elgin and Reuter 2013).

**Fig. 7.**
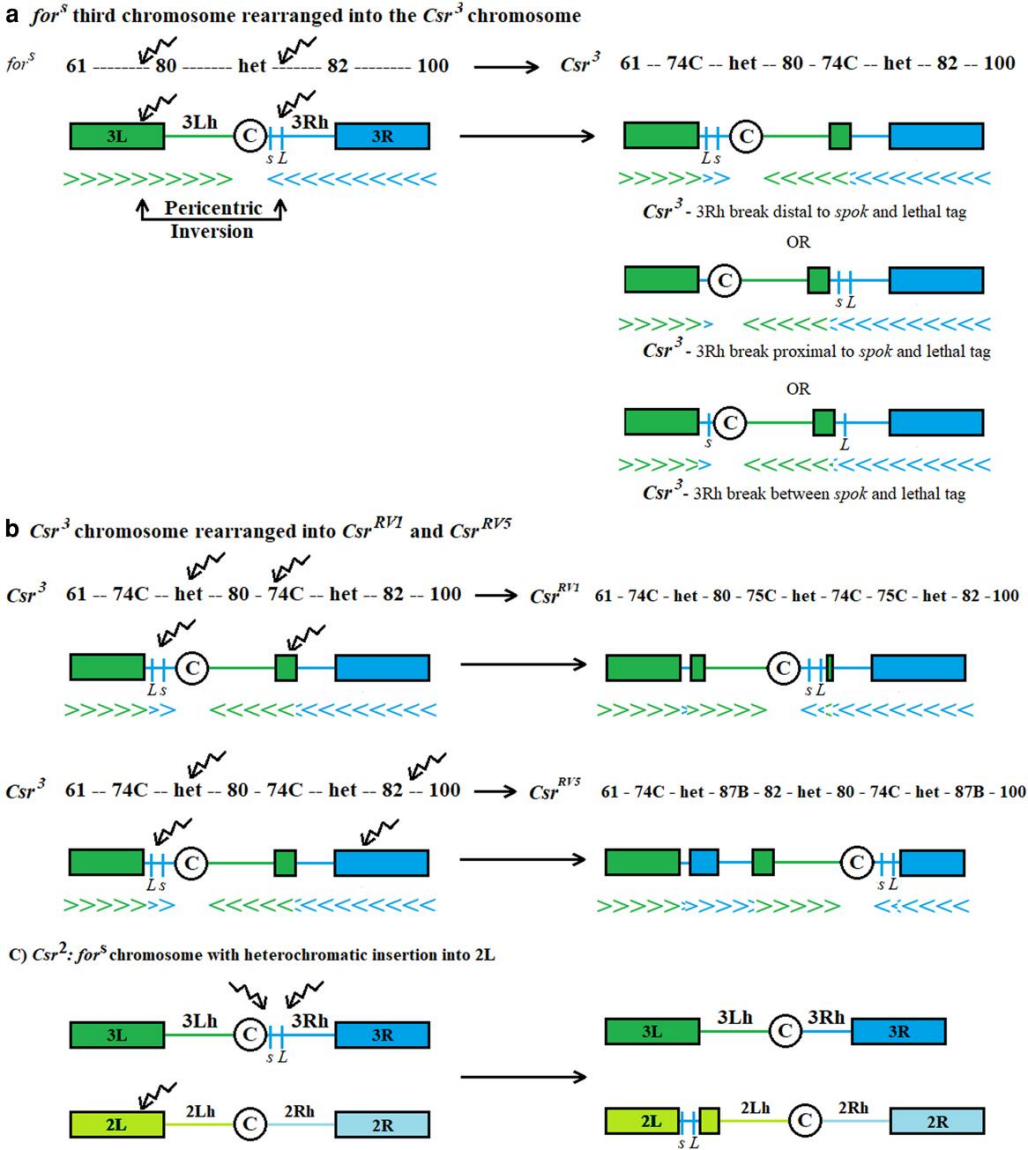
Visual representation of *D. melanogaster* chromosomes showing possible rearrangements causing *spok* to variegate. C is the centromere, *s* represents *spok* and *L* represents the pupal lethal tag. Numbers above the chromosome represent cytological bands. Squiggly arrows show the location of X-ray induced chromosome breaks determined by polytene analysis. a) Creation of the *Csr^3^* chromosome from a simple pericentric inversion in *for^s^*. b) Creation of the *Csr^RV1^* and *Csr^RV5^* chromosomes from *Csr^3^*. c) Creation of the *Csr^2^* chromosome by a direct insertion into 2L euchromatin.

With PEV, it is often the case that there are no aberrations within the coding region of the variegating gene, as it is the change in the chromatin environment that alters gene expression. In line with this, we did not find convincing evidence of any potentially significant coding region changes in *Csr* except for a single nucleotide polymorphism that originated in the *Csr^3^* line. This polymorphism was also present the *Csr^RV^* lines as they were derived from *Csr^3^*, but the *Csr^RV^* lines did not possess any other alterations in *spok* despite having been through an additional x-ray mutagenesis. It was likely that the large mutagenesis screens conducted by Pereira *et al*. (1995) did produce direct hits to *spok*, but *spok* mutations lead to issues with larval molting, with null *spok* larvae failing to progress past the 1st instar stage (Ono *et al*. 2006). Consequently, it would have been unlikely to recover direct hits to *spok* in the Pereira *et al*. (1995) screen, which selected third instar larvae. Figure 7 provides a visual representation of how some of the chromosomal rearrangements in the *Csr* lines could lead to variegation in the *spok* gene and the pupal lethal tag. Because heterochromatin is not polytenized in salivary gland chromosomes, the exact extent of X-ray damage to heterochromatin could not be determined so there could be multiple undetectable breaks within heterochromatin in any *Csr* line. Despite the uncertainty surrounding the number of heterochromatic breaks, we know that *Csr^3^* could be variegating in the *Csr* lines for several reasons: *Csr* was moved from its position deep within proximal heterochromatin to a position in distal euchromatin, and/or a rearrangement brought a block of euchromatin in close proximity to *Csr*. In Fig. 7, visual representations of chromosome rearrangements were made using the fewest heterochromatic breaks needed to produce the chromosome order established in the polytene chromosome analyses. Additionally, in Fig. 7, the pupal lethal tag and *spok* are shown to move together in the creation of *Csr^RV1^* and *Csr^RV5^*, but it is possible that a rearrangement breakpoint occurred between *spok* and the lethal tag, separating them onto different arms.

Deletion mapping of heterochromatin reduced our candidate region for *Csr* to 2 essential genes in 3Rh, *PARP1* and *spok*, and 2 final pieces of evidence confirmed that *Csr* mapped to *spok*. Firstly, qRT-PCR demonstrated that *spok* expression levels differed between rovers (*for^R^*) and sitters (*for^s^*), with rover expression higher than sitter. Secondly, feeding 20E ecdysone hormone to *for^s^* larvae at a concentration shown not to effect developmental time, changed the behavior of sitter larvae into rovers; the same behavioral change produced by *Csr*. Although we would theoretically expect the sitter-like *Csr^RV^* lines to also change behavior when fed 20E hormone, the *Csr^RV^* lines were not tested for behavior because they were designed as mapping tools. Specifically, the chromosomes in these lines had undergone 2 successive rounds of X-ray mutagenesis producing extremely complex chromosome rearrangements. The sheer number of potential secondary effects in the *Csr^RV^* lines made them poor candidates for our 20E feeding experiments. Although not shown, during our search for *Csr* through the entirety of chromosome 3 euchromatin we found numerous secondary site effects ranging from full embryonic lethality, bristle abnormalities, and headless pharate pupae. These secondary effects were limited to individual *Csr^RV^* lines and therefore did not map to *Csr.*

Mapping *Csr* to the *spok* gene creates a link between ecdysone signaling and larval foraging behavior. There are known ecdysone pulses during larval development of *D. melanogaster* (Riddiford and Truman 1993; Warren *et al*. 2006). For example, pulses in the first and second larval instar trigger molting of the larva and there are 3 low-titre pulses in the third instar that are associated with critical weight, glue secretion and the switch from foraging behavior to wandering behavior, when larvae leave the food in search of a pupation site (Morrow and Mirth 2024). Mapping the *Csr* gene to *spok*, an ecdysone signaling gene, provides the first links between larval foraging behavior, ecdysone signaling and the *foraging* gene, a cGMP dependent protein kinase (PKG) of *D. melanogaster.* Insights from other invertebrates suggest that PKG acts in the signaling pathways associated with ecdysteroid synthesis in the molting glands of decapod crustaceans (Mykles 2021). Iino *et al.* (2023) report that certain targets of ecdysone signaling genes are upregulated during foraging behavior in the adult honeybee worker brain, a time when the honeybee *foraging* gene, *Amfor*, is also known to be upregulated (Ben-Shahar *et al*. 2002).

In hindsight, the lethal tagging screen by Pereira *et al*. (1995) was extremely successful, but at the time, the study of heterochromatin as a functional domain of gene expression was in its infancy and there were very few tools available to map genes within heterochromatin. It was reasonable at the time to begin our search for *Csr* by attempting to map the original temperature sensitive pupal lethal tag in the euchromatic candidate region originally established by Pereira *et al*. (1995), but once we could not find evidence of *Csr* in this region we hypothesized that *Csr* likely shared developmental pathways with genes in the area (specifically *jar* and *crb*). Discovering that *Csr* was variegating in manner consistent with a heterochromatic gene supported this hypothesis and eliminated the *jar-crb* region as a candidate region. Links between ecdysone signaling and both *jar* and crb have since been established. Some of the shared pathways include *jar* being shown to regulate ecdysone signaling, and both ecdysone and *crb* contributing to the complex *Hippo* signaling pathway (Ling *et al*. 2010; Yoo *et al*. 2021; Fu *et al*. 2022). It was these links to ecdysone signaling that initially misled the mapping of *Csr* to the *jar*-*crb* region when mapping tools for heterochromatin were limited.

Adding to the list of roadblocks faced by the initial efforts to map *Csr* was the simple fact that the *spok* gene itself, like most heterochromatic genes at the time, had not been characterized, nor was it even a predicted coding region; Drosophila heterochromatin had not yet been sequenced. *spok* was first classified as a putative truncated pseudo gene by Tijet *et al*. in 2001 and *spok* was confirmed as a protein coding gene by Ono *et al*. (2006), long after *Csr* was first characterized. In the end, lethal tagging was successful, and a great method for mapping *Csr* as a dominant modifier of larval foraging behavior, but Pereira *et al*. (1995) were unknowingly ahead of their time in trying to study and map an essential heterochromatic gene.

## Supplementary Material

iyaf123_Supplementary_Data

## Data Availability

Data generated or analyzed during this study are provided in full within the published article and its Supplementary materials. Supplemental material available at GENETICS online.
