# Supplementary material for: Alleles of Chaser, a dominant modifier of the Drosophila melanogaster foraging gene, are consistent with variegating alleles of the heterochromatic gene spookier
Source: Genetics. 2025 Jun 26;231(1):iyaf123. doi: 10.1093/genetics/iyaf123 (PMC12406005; doi:10.1093/genetics/iyaf123)

Table S1. Complementation matrix of *D. melanogaster* deletions in cytological band 95F showing all *inter se* combinations. A minus sign denotes a failure to complement and a positive sign indicates the two deletions complemented one another.

|  | *Df(3R)crb87-4* | *Df(3R)crb87-5* | *Df(3R)*w5 | *Df(3R)*6198 | *Df(3R)*6199 | *Df(3R)8178* |
| --- | --- | --- | --- | --- | --- | --- |
| *Df(3R)crb*87-4 | - |  |  |  |  |  |
| *Df(3R)crb*87-5 | - | - |  |  |  |  |
| *Df(3R)*w5 | - | + | - |  |  |  |
| *Df(3R)*6198 | - | - | - | - |  |  |
| *Df(3R)*6199 | - | - | + | + | - |  |
| *Df(3R)8178* | - | - | + | + | - | - |

Table S2. Sample progeny counts from the complementation matric in Table S1. TM3 refers to progeny that have a TM3 balancer chromosome [containing the bristle marker *Stubble* (*Sb*) and the wing marker *Serrate* (*Ser*)]

| Deletion 1 | Deletion 2 | n | TM3 | TM3^+^ |
| --- | --- | --- | --- | --- |
| *Df(3R)crb*87-4 | *Df(3R)crb*87-5 | 86 | 86 | 0 |
| *Df(3R)crb*87-4 | *Df(3R)*w5 | 135 | 131 | 4 |
| *Df(3R)crb*87-4 | *Df(3R)*6198 | 74 | 74 | 0 |
| *Df(3R)crb*87-4 | *Df(3R)*6199 | 75 | 75 | 0 |
| *Df(3R)crb*87-4 | *Df(3R)8178* | 68 | 68 | 0 |
| *Df(3R)crb*87-5 | *Df(3R)*w5 | 135 | 96 | 39 |
| *Df(3R)crb*87-5 | *Df(3R)*6198 | 104 | 103 | 1 |
| *Df(3R)crb*87-5 | *Df(3R)*6199 | 119 | 119 | 0 |
| *Df(3R)crb*87-5 | *Df(3R)8178* | 135 | 135 | 0 |
| *Df(3R)*w5 | *Df(3R)*6198 | 120 | 120 | 0 |
| *Df(3R)*w5 | *Df(3R)*6199 | 99 | 62 | 37 |
| *Df(3R)*w5 | *Df(3R)8178* | 111 | 68 | 43 |
| *Df(3R)*6198 | *Df(3R)*6199 | 112 | 68 | 44 |
| *Df(3R)*6198 | *Df(3R)8178* | 96 | 69 | 27 |
| *Df(3R)*6199 | *Df(3R)8178* | 81 | 81 | 0 |

Table S3. Sample progeny counts for *D. melanogaster* crosses between deletions in cytological band 95F and the *Csr^RV^* lines at 29^o^C. TM3 refers to progeny that have a TM3 balancer chromosome [containing the bristle marker *Stubble* (*Sb*) and the wing marker *Serrate* (*Ser*)]. TM3^+^ refer to heterozygous unbalanced progeny and n is the total progeny scored per cross.

| Deletion | *Csr^RV#^* | n | TM3 | TM3^+^ |  | Deletion | *Csr^RV#^* | n | TM3 | TM3^+^ |
| --- | --- | --- | --- | --- | --- | --- | --- | --- | --- | --- |
| *Df(3R)crb*87-4 | *Csr^RV1^* | 75 | 75 | 0 |  | *Df(3R)crb*87-5 | *Csr^RV1^* | 55 | 54 | 1 |
| *Df(3R)crb*87-4 | *Csr^RV2^* | 56 | 0 | 0 |  | *Df(3R)crb*87-5 | *Csr^RV2^* | 74 | 74 | 0 |
| *Df(3R)crb*87-4 | *Csr^RV3^* | 88 | 70 | 18 |  | *Df(3R)crb*87-5 | *Csr^RV3^* | 50 | 43 | 7 |
| *Df(3R)crb*87-4 | *Csr^RV4^* | 80 | 69 | 11 |  | *Df(3R)crb*87-5 | *Csr^RV4^* | 68 | 66 | 2 |
| *Df(3R)crb*87-4 | *Csr^RV5^* | 76 | 61 | 15 |  | *Df(3R)crb*87-5 | *Csr^RV5^* | 79 | 72 | 7 |
| *Df(3R)crb*87-4 | *Csr^RV6^* | 57 | 51 | 6 |  | *Df(3R)crb*87-5 | *Csr^RV6^* | 57 | 51 | 6 |
| *Df(3R)crb*87-4 | *Csr^RV7^* | 57 | 50 | 7 |  | *Df(3R)crb*87-5 | *Csr^RV7^* | 69 | 62 | 7 |
|  |  |  |  |  |  |  |  |  |  |  |
| *Df(3R)*w5 | *Csr^RV1^* | 138 | 77 | 61 |  | *Df(3R)*6199 | *Csr^RV1^* | 79 | 52 | 27 |
| *Df(3R)*w5 | *Csr^RV2^* | 107 | 65 | 42 |  | *Df(3R)*6199 | *Csr^RV2^* | 100 | 89 | 11 |
| *Df(3R)*w5 | *Csr^RV3^* | 103 | 65 | 38 |  | *Df(3R)*6199 | *Csr^RV3^* | 95 | 63 | 32 |
| *Df(3R)*w5 | *Csr^RV4^* | 111 | 68 | 43 |  | *Df(3R)*6199 | *Csr^RV4^* | 96 | 57 | 39 |
| *Df(3R)*w5 | *Csr^RV5^* | 83 | 52 | 31 |  | *Df(3R)*6199 | *Csr^RV5^* | 101 | 70 | 31 |
| *Df(3R)*w5 | *Csr^RV6^* | 112 | 48 | 64 |  | *Df(3R)*6199 | *Csr^RV6^* | 130 | 56 | 74 |
| *Df(3R)*w5 | *Csr^RV7^* | 101 | 62 | 39 |  | *Df(3R)*6199 | *Csr^RV7^* | 105 | 69 | 36 |
|  |  |  |  |  |  |  |  |  |  |  |
| *Df(3R)*6198 | *Csr^RV1^* | 84 | 52 | 32 |  | *Df(3R)*8178 | *Csr^RV1^* | 72 | 53 | 19 |
| *Df(3R)*6198 | *Csr^RV2^* | 89 | 64 | 5 |  | *Df(3R)*8178 | *Csr^RV2^* | 58 | 55 | 3 |
| *Df(3R)*6198 | *Csr^RV3^* | 80 | 50 | 30 |  | *Df(3R)*8178 | *Csr^RV3^* | 116 | 78 | 38 |
| *Df(3R)*6198 | *Csr^RV4^* | 123 | 75 | 48 |  | *Df(3R)*8178 | *Csr^RV4^* | 113 | 74 | 39 |
| *Df(3R)*6198 | *Csr^RV5^* | 77 | 55 | 22 |  | *Df(3R)*8178 | *Csr^RV5^* | 82 | 57 | 25 |
| *Df(3R)*6198 | *Csr^RV6^* | 93 | 50 | 43 |  | *Df(3R)*8178 | *Csr^RV6^* | 79 | 30 | 49 |
| *Df(3R)*6198 | *Csr^RV7^* | 80 | 52 | 28 |  | *Df(3R)*8178 | *Csr^RV7^* | 113 | 70 | 43 |

Table S4. Sample progeny counts for *D. melanogaster* crosses between deletions in cytological band 95F and *jar* and *crb* alleles, at 29^o^C. TM3 refers to progeny that have a TM3 balancer chromosome [containing the bristle marker *Stubble* (*Sb*) and the wing marker *Serrate* (*Ser*)]. TM3^+^ refer to heterozygous unbalanced progeny and n is the total progeny scored per cross.

| Deletion | *Allele* | n | TM3 | TM3^+^ |  | Deletion | *Allele* | n | TM3 | TM3^+^ |
| --- | --- | --- | --- | --- | --- | --- | --- | --- | --- | --- |
| *Df(3R)crb*87-4 | *crb^S010409^* | 97 | 97 | 0 |  | *Df(3R)crb*87-5 | *crb^S010409^* | 108 | 108 | 0 |
| *Df(3R)crb*87-4 | *crb^07207^* | 108 | 108 | 0 |  | *Df(3R)crb*87-5 | *crb^07207^* | 51 | 51 | 0 |
| *Df(3R)crb*87-4 | *crb^j1b5^* | 78 | 78 | 0 |  | *Df(3R)crb*87-5 | *crb^j1b5^* | 120 | 120 | 0 |
| *Df(3R)crb*87-4 | *jar^2095^* | 74 | 74 | 0 |  | *Df(3R)crb*87-5 | *jar^2095^* | 109 | 109 | 0 |
| *Df(3R)crb*87-4 | *jar^1646^* | 91 | 91 | 0 |  | *Df(3R)crb*87-5 | *jar^1646^* | 151 | 151 | 0 |
|  |  |  |  |  |  |  |  |  |  |  |
| *Df(3R)*w5 | *crb^S010409^* | 99 | 63 | 36 |  | *Df(3R)*6199 | *crb^S010409^* | 128 | 128 | 0 |
| *Df(3R)*w5 | *crb^07207^* | 111 | 80 | 31 |  | *Df(3R)*6199 | *crb^07207^* | 103 | 103 | 0 |
| *Df(3R)*w5 | *crb^j1b5^* | 168 | 118 | 50 |  | *Df(3R)*6199 | *crb^j1b5^* | 59 | 59 | 0 |
| *Df(3R)*w5 | *jar^2095^* | 137 | 137 | 0 |  | *Df(3R)*6199 | *jar^2095^* | 88 | 54 | 34 |
| *Df(3R)*w5 | *jar^1646^* | 69 | 69 | 0 |  | *Df(3R)*6199 | *jar^1646^* | 95 | 57 | 38 |
|  |  |  |  |  |  |  |  |  |  |  |
| *Df(3R)*6198 | *crb^S010409^* | 79 | 57 | 22 |  | *Df(3R)*8178 | *crb^S010409^* | 109 | 109 | 0 |
| *Df(3R)*6198 | *crb^07207^* | 78 | 50 | 28 |  | *Df(3R)*8178 | *crb^07207^* | 162 | 162 | 0 |
| *Df(3R)*6198 | *crb^j1b5^* | 80 | 55 | 25 |  | *Df(3R)*8178 | *crb^j1b5^* | 127 | 127 | 0 |
| *Df(3R)*6198 | *jar^2095^* | 71 | 71 | 0 |  | *Df(3R)*8178 | *jar^2095^* | 81 | 59 | 22 |
| *Df(3R)*6198 | *jar^1646^* | 86 | 86 | 0 |  | *Df(3R)*8178 | *jar^1646^* | 84 | 53 | 31 |

Table S5. Data showing that the *D. melanogaster* *crb^COS-P1^* cosmid completely rescues the abnormal abdominal banding phenotype observed in *Csr*^RV2^ / *crb* heterozygotes. TM3 refers to progeny that have a TM3 balancer chromosome [containing the bristle marker *Stubble* (*Sb*) and the wing marker *Serrate* (*Ser*)]. TM3^+^ refer to heterozygous unbalanced progeny.

| Cross at 25°C | # TM3 progeny | # TM3^+^ progeny | TM3^+^ progeny with Abdominal Banding Abnormalities |
| --- | --- | --- | --- |
| *crb^COS-P1^* ; *Csr*^RV2^ / TM3 x *crb^06985^* | 67 | 43 | None |
| *crb^COS-P1^* ; *Csr*^RV2^ / TM3 x *crb^S010409^* | 76 | 38 | None |
| *Csr*^RV2^ / TM3 x *crb^06985^* | 49 | 23 | 18 |
| *Csr*^RV2^ / TM3 x *crb^S010409^* | 67 | 24 | 22 |

Table S6. Data showing that the *D. melanogaster crb^COS-P1^* cosmid does not rescue the temperature sensitive pupal lethal tag. . TM3 refers to progeny that have a TM3 balancer chromosome [containing the bristle marker *Stubble* (*Sb*) and the wing marker *Serrate* (*Ser*)]. TM3^+^ refer to heterozygous unbalanced progeny.

| Cross at 29°C | # TM3 progeny | # TM3^+^ progeny |
| --- | --- | --- |
| *crb^COS-P1^* x *crb^COS-P1^* | 0 | 94 |
| *Csr*^RV2^ / TM3 x *Csr*^RV1^ / TM3 | 76 | 0 |
| *crb^COS-P1^* ; *Csr*^RV2^ / TM3 x *crb^COS-P1^* ; *Csr*^RV1^ | 44 | 2 |
| *Csr*^RV1^ / TM3 x *Csr*^RV3^ / TM3 | 157 | 0 |
| *crb^COS-P1^* ; *Csr*^RV1^ / TM3 x *crb^COS-P1^* ; *Csr*^RV3^ | 37 | 0 |
| *Csr*^RV3^ / TM3 x *Csr*^RV5^ / TM3 | 141 | 0 |
| *crb^COS-P1^* ; *Csr*^RV3^ / TM3 x *crb^COS-P1^* ; *Csr*^RV5^ | 52 | 0 |
| *Csr*^RV2^ / TM3 x *Csr*^RV3^ / TM3 | 42 | 0 |
| *crb^COS-P1^* ; *Csr*^RV2^ / TM3 x *crb^COS-P1^* ; *Csr*^RV3^ | 76 | 3 |
| *Csr*^RV1^ / TM3 x *Csr*^RV5^ / TM3 | 62 | 0 |
| *crb^COS-P1^* ; *Csr*^RV1^ / TM3 x *crb^COS-P1^* ; *Csr*^RV5^ | 51 | 0 |

**Figure S1. Deletion map of the *D. melanogaster jar-crb* region contains nine known or predicted coding regions.** This *jar-crb* region deletion map was drawn from the complementation data in supplementary Table S1 and S2 combined with cytological data available on FlyBase (http://flybase.org). The thick horizontal line represents the chromosome, the arrows represent known or predicted coding regions (arrows point in the direction of transcription), and solid horizontal lines below the genes represent deleted regions in deficiencies. The breakpoints of the deficiencies are not exact since they are based on cytological data, so the approximate location of the breakpoints are marked by dashed lines.


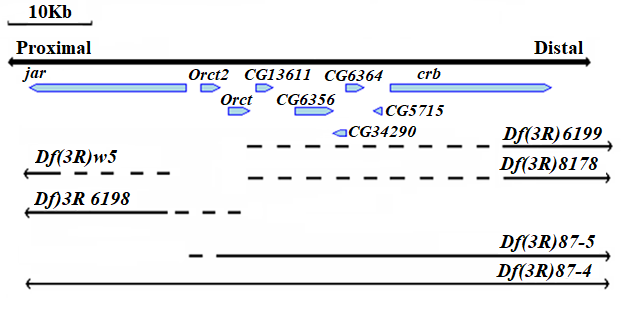


Figure S2. Hand drawings of wing vein phenotypes of varying combination of *jar* mutants (*jar*^1646^ and *jar*^2095^), *Csr* mutants (*Csr*^RV2^ and *Csr*^3^), and deletions in the *jar-crb* region (*w5 and 87-5, and F89-4*). The larger top wing is wildtype.


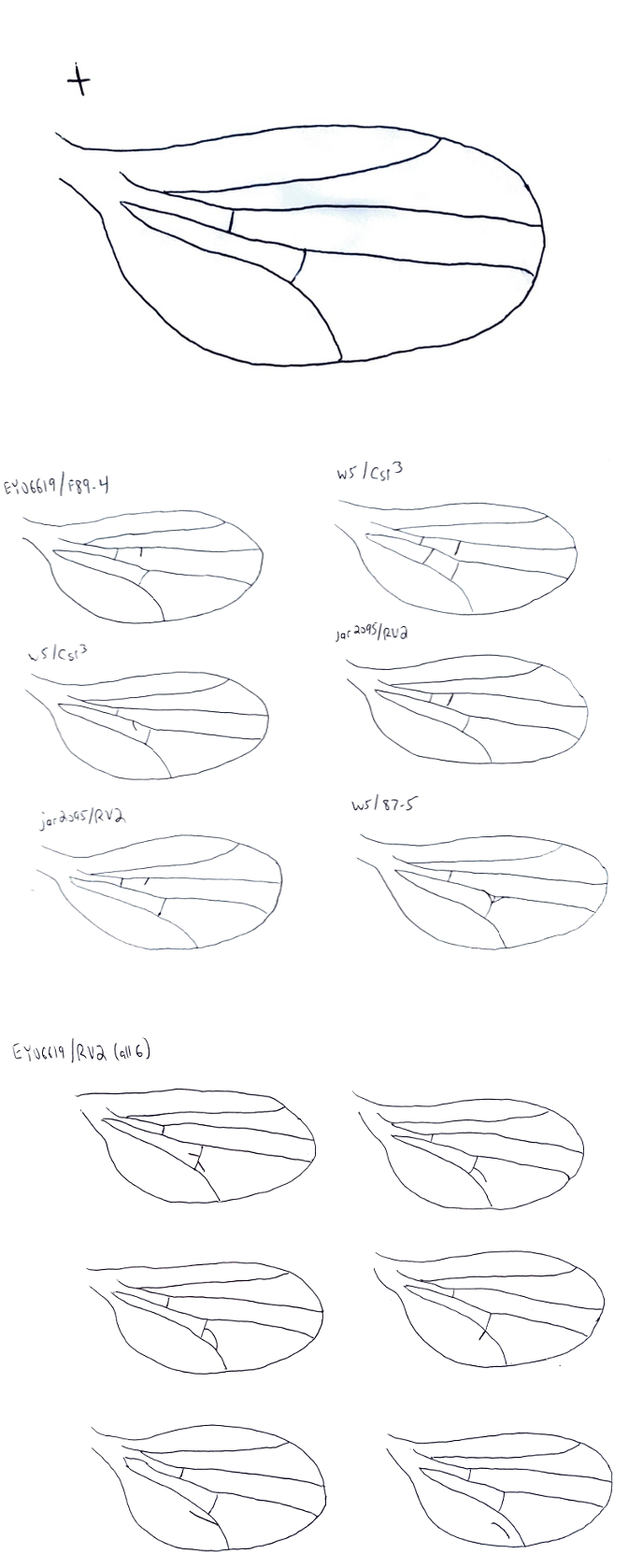

Supplement: iyaf123_Supplementary_Data [file iyaf123_supplementary_data.docx]
